# Outcomes in Adults with Celiac Disease Following a Gluten-Free Diet

**DOI:** 10.3390/jcm14145144

**Published:** 2025-07-20

**Authors:** Daniel Vasile Balaban, Iulia Enache, Marina Balaban, Răzvan Andrei David, Andreea-Diana Vasile, Alina Popp, Mariana Jinga

**Affiliations:** 1 Faculty of Medicine, University of Medicine and Pharmacy Carol Davila, 050474 Bucharest, Romania; vasile.balaban@umfcd.ro (D.V.B.); iulia.enache@drd.umfcd.ro (I.E.); marina.balaban@yahoo.com (M.B.); razvandavid1919@gmail.com (R.A.D.); mariana.jinga@umfcd.ro (M.J.); 2 Central Military Emergency University Hospital Dr. Carol Davila, 010242 Bucharest, Romania; 3 National Institute for Mother and Child Health “Alessandrescu-Rusescu”, 020382 Bucharest, Romania; sprinceanadiana@yahoo.com; 4 Celiac Disease Research Center, Faculty of Medicine and Health Technology, Tampere University and Tampere Hospital, 33520 Tampere, Finland

**Keywords:** celiac disease, follow-up, histology, remission, recovery, persistent, villous atrophy, GFD-treated

## Abstract

**Background/Objectives**: Histological follow-up still lacks consensus in the long-term management of adult patients with celiac disease (CD) adhering to a gluten-free diet (GFD). Despite clinical and serological improvement, a significant proportion of patients continue to have persistent villous atrophy. We aimed to synthesize current evidence regarding histological outcomes after GFD treatment in adult CD, focusing on mucosal healing rates, assessment methods, and remission criteria. **Methods**: We conducted a literature search with extraction and analysis of published cohort studies that included adult patients with CD on GFD with follow-up biopsy data. Extracted parameters included demographic details, baseline histology, GFD duration and adherence, serologic status, and histologic recovery rates with corresponding remission criteria. **Results**: Data from 46 studies comprising 15,530 patients were analyzed. The overall mean age was 41 years, and 73.3% were female. Mean histologic remission across cohorts was 58.8%, with considerable interstudy variation. Remission criteria also varied widely, ranging from strict Marsh 0 control histology to more inclusive definitions that considered Marsh 1 or even non-atrophic mucosa (Marsh < 3) as indicative of recovery, while some studies relied on quantitative villous height-to-crypt depth ratio thresholds, substantially influencing reported remission rates. Longer GFD duration and rigorous diet adherence assessment using validated questionnaires and accurate laboratory tools were associated with higher remission rates. **Conclusions**: Histologic remission in GFD-treated adult patients with CD is highly variable and strongly influenced by remission definitions and adherence assessment methods. Standardized reporting using validated metrics for histologic outcome and dietary compliance is essential for harmonizing follow-up strategies in adult CD.

## 1. Introduction

Celiac disease (CD) is a chronic immune-mediated enteropathy with systemic involvement, triggered by gluten ingestion in genetically predisposed individuals. With a global prevalence of about 1%, CD is a common but severely underdiagnosed chronic digestive disease [1]. The consequence of gluten-induced autoimmune phenomena in CD is represented by small bowel mucosal injury, consisting of villous atrophy (VA), crypt hyperplasia, and intraepithelial inflammation. Diagnosis of adult CD currently relies on a combination of serological testing (anti-tissue transglutaminase, deamidated gliadin peptide, and endomysial antibodies) and endoscopy with small bowel biopsies revealing atrophic mucosal damage [2]. The only effective treatment for CD remains a strict, lifelong gluten-free diet (GFD), which typically improves symptoms and leads to progressive negative seroconversion. Dietary elimination of gluten, the pathogenic trigger in CD, interrupts the underlying autoimmune cascade, enabling clinical recovery and the gradual restoration of villous architecture. However, complete histological recovery of the bowel mucosa lags behind clinical remission, and persistent lesions may occur despite good adherence to the diet [3]. In the setting of incomplete mucosal recovery, there is a growing interest in the development of non-nutritional therapies for CD, aiming at achieving full histologic remission and improving long-term outcomes [4].

As for other chronic inflammatory, immune-mediated bowel diseases, such as inflammatory bowel diseases (Crohn’s disease and ulcerative colitis), mucosal healing is ALSO the goal in CD [5]. Histologic remission has been proven to decrease the major risk linked with CD, that of lymphoproliferative malignancy [6], and has also been associated with reduced mortality [7]. While there has been increasing interest in finding surrogate biomarkers that correlate with reversal of mucosal damage, histology remains the gold standard to document intestinal recovery in individuals with CD. Symptom resolution is not a reliable predictor of mucosal status, and normalization of serologic markers also lacks sensitivity in detecting persistent VA [8]. Likewise, correlation of other laboratory markers with duodenal histology remains modest [3,9].

In this setting, follow-up biopsy remains the most reliable tool to demonstrate restitution of bowel mucosa in GFD-treated CD individuals. However, there is no consensus on the routine use of control biopsy in adults with CD, and currently available guidelines do not universally recommend a follow-up biopsy in patients with good adherence and response to GFD, clinically and serologically [2]. While some authors argue against a repeat biopsy in well-controlled patients, which is invasive and does not impact patient management, others advocate its use to identify at-risk patients for complications such as refractory CD as well as to check for mucosal recovery in patients with seronegative CD [3]. Moreover, there is also a lack of consensus with regard to the timing of biopsy (as mucosal healing rates improve over time), a tailored approach according to baseline severity of intestinal damage (as recovery is faster in milder enteropathy), patient-related risk factors (for associated pathology that might be detected during endoscopy), or special phenotypes such as “slow-responders” [10]. Also, there is heterogeneity in defining histologic recovery in adult CD; some authors consider it as Marsh 0 appearance of bowel mucosa, others as Marsh 0–1, and others as restitution of villous morphology. Among the histologic criteria studied in GFD-treated CD individuals, persistent intraepithelial lymphocytosis has been commonly reported despite long-term strict adherence to the diet [11,12].

In the present study, we aimed to synthesize existing data on histologic remission in adult CD cohorts treated with GFD to better delineate the rates of mucosal healing according to clinical and laboratory correlates and to critically evaluate the role of follow-up biopsy in the long-term management of adult CD.

## 2. Methodology

### 2.1. Literature Search

For the purpose of this scoping review, a literature search was performed in December 2024 using dedicated search strings (Supplementary File S1), which returned 257 records on PubMed and 439 on Scopus. Original articles with English abstracts available were further screened for relevance to the topic of our research. We included papers referring to adult CD with follow-up histology on GFD. Exclusion criteria were pediatric CD, refractory CD, and articles without English full text. We did not analyze pediatric CD, as histologic remission rates and dynamics are different from adult CD. Also, baseline histology has not been required in children since the 2012 ESPGHAN guideline [13], and follow-up histology is not routinely conducted in this patient population. Refractory CD was also excluded, as it implies, by definition, persistence of VA despite strict adherence to GFD.

### 2.2. Study Selection

Altogether, 33 studies were selected after the abstract screening stage, which were analyzed in full text for eligibility. Of them, two were excluded because of inadequate study population or insufficient results: Wahab et al. [14], as the cohort comprised both adults and children and the histologic remission rates were presented for the entire group, not allowing for adult subgroup analysis, and Singh et al. [15], as it referred to patients with minimal enteropathy (Marsh 1 lesions). Other studies that referred to both pediatric and adult CD but provided data for mucosal healing in subgroups, such as Bardella et al. [16], were included in the final analysis. Another 3 articles were excluded because they were old studies referring to jejunal biopsies, as it would have represented an additional element of heterogeneity [17,18,19]. Another paper initially considered eligible was also excluded from analysis as it reported on data from a clinical trial [20].

The remaining 27 studies were considered eligible for inclusion and processed for data extraction in duplicate by two independent investigators in a standardized Excel form, with disagreements resolved by discussion and consensus. Another 23 relevant papers were added to the final analysis by backward and forward reference search, also by other sources that came to the attention of investigators (e.g., research platforms). Some older studies without precise quantification of histologic remission were not included [21,22]. Moreover, from this second round of potentially eligible studies, there were another 2 exclusions: Patel et al. [23], comprising a cohort of both pediatric and adult patients with CD, was excluded because quantitative histologic outcomes were not reported separately for adults, and Annibale et al. [24] was excluded because the study did not report the proportion of patients achieving mucosal recovery, only noting the correlation of histological score of duodenitis with anemia resolution.

**Figure 1:**
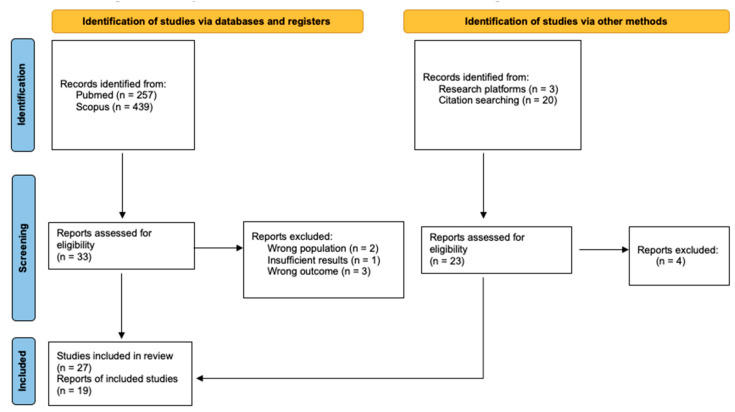
Flowchart of study identification, screening, and selection.

The flowchart depicting study selection for this review is illustrated in Figure 1. About half of the included studies were prospective in design, incorporating standardized follow-up protocols or serial assessments of bowel histology at diagnosis and after GFD. One study was classified as ambispective, a few others were retrospective analyses of prospectively maintained databases, and the remaining ones were retrospective in nature. In terms of study type, most were observational, either cross-sectional or follow-up (longitudinal), while few were diagnostic accuracy studies that included data on follow-up histology.

### 2.3. Data Extraction and Processing

Parameters extracted from selected studies were as follows: study first author and year of publication, population studied, number of CD patients included, gender representation, histology at diagnosis, duration of GFD, tools used for assessment of GFD adherence, proportion of histologic remission, criteria used to evaluate histologic remission, and, if available, proportion of patients with negative serology and asymptomatic. When exact data was not available for the required parameters, estimations were carried out if statistically possible, and results were marked in the summarizing table as “estimated”. For example, in studies in which demographic data such as median age were only reported for subgroups, overall cohort estimates were calculated using a weighted average approach [11,25,26,27,28]. Specifically, we calculated the weighted median age and female percentage by multiplying the reported subgroup values by their corresponding sample sizes, adding these products, and dividing the sum by the total cohort size. Weighted averages were also computed for other parameters when subgroup data was available, such as proportion of histologic remission [25] or asymptomatic patients [29]. Also, in studies where gender proportion was reported as male/female, the proportion of female participants was derived by considering the total number of individuals with CD [30]. Also, when papers did not explicitly report the rate of histologic remission but the proportion of patients with persistent VA was available, we counted the remaining as mucosal recovery [31]. Similarly, when indirect data was reported, the metric required for analysis was calculated as the difference from 100, e.g., the proportion of patients without seroconversion [32]. When data was available, the proportion of histologic remission was reported to the number of patients with strict adherence to GFD and not to the entire cohort [32,33].

### 2.4. Statistical Analysis

Descriptive statistics was used to summarize histologic outcomes in GFD-treated adults with CD. Continuous variables were reported using median and range (minimum–maximum), while categorical variables were expressed as absolute counts and percentages. Statistical analysis was carried out using Microsoft Excel version 16.29 and SPSS version 20.

## 3. Results

### 3.1. Patient Demographics

Altogether, 46 studies were included, encompassing published data for 15,530 CD patients over a timespan of more than two decades, from 1998 to 2024. Most papers reported biopsy-proven CD as inclusion criteria, but there was heterogeneity with regard to histology at diagnosis: the majority of studies used Marsh classification and included patients with Marsh 3 mucosal injury, while some included Marsh ≥ 2 with positive serology. The summary of data collected from included studies is presented in Table 1 [7,11,16,25,26,27,28,29,30,31,32,33,34,35,36,37,38,39,40,41,42,43,44,45,46,47,48,49,50,51,52,53,54,55,56,57,58,59,60,61,62,63,64,65,66,67].

Reporting of patient demographics was also not uniform: while most studies presented age as median (years), accompanied by interquartile range, some reported it as mean ± standard deviation [29], and others only as range (min–max) [55]. Subject to this uneven presentation of the data, the mean age was 41 years. With regard to gender distribution, there was definite female predominance among all studies, with overall 73.3% of CD patients being women.

### 3.2. GFD Duration and Assessment

The duration of the GFD prior to follow-up histologic assessment varied substantially across the included studies. Most commonly, patients underwent repeat biopsy after at least 12 months of GFD, with 1-year or 2-year durations being the most frequent fixed follow-up intervals for histological reassessment. Similarly to other parameters, we also noted heterogenous reporting in GFD duration: while most studies reported median (range) of GFD, some presented data as mean ± SD [35,38], others as minimal threshold duration (e.g., GFD ≥ 1 year), and others as split patients into subgroups (short-term-treated vs. long-term-treated) [50,60,66]. There was significant time range variability, with cohorts reporting wide ranges of GFD durations, certain studies referring to diet adoption for a fixed follow-up timepoint of 1 or 2 years, and a small number of studies investigating long-term-treated patients with CD beyond a decade [28,42,48].

While longer durations of GFD were generally associated with improved mucosal healing, this observed trend is counterbalanced by data showing that histologic remission was not guaranteed by time passing alone. Notably, some studies with follow-up periods exceeding 5 years still reported persistent VA in a sizeable proportion of patients [33,53]. Conversely, high rates of mucosal recovery were observed in some studies even after relatively short durations of GFD (1–2 years) [25,45], particularly with rigorous adherence assessment using f-GIP (fecal gluten immunogenic peptides) as in Fernández-Bañares et al. [45]. These findings underscore the heterogeneity of mucosal recovery timelines, reflecting on slow vs. fast responders on GFD, the use of accurate tools to monitor adherence versus self-declared diet compliance, lack of correlation between diet duration and mucosal healing, and the delineation of a potential subset of patients that require more intensive follow-up strategies due to persistence of mucosal lesions.

Regarding GFD adherence, assessment was highly heterogeneous across the included papers. While some studies relied on serologic testing, most used qualitative dietary assessment by means of dietitian/physician interview, food diaries, or structured tools (Biagi score, CDAT—Celiac Dietary Adherence Test), and only a minority used detection of biomarkers such as GIP (gluten immunogenic peptides), namely, f-GIP in one study [45] and u-GIP (urinary gluten immunogenic peptides) in two [25,36]. Despite being a validated and reproducible instrument, the CDAT was only used in five studies. Only a few studies provided no adherence evaluation or weak assessments, such as patient self-reported compliance, introducing potentially significant bias in the interpretation of histologic outcomes. Conversely, few studies used composite approaches to evaluate GFD adherence, integrating clinical follow-up, serologic testing, and dietetic input.

### 3.3. Histological Remission Criteria

The Marsh–Oberhuber classification is the current standard for reporting histologic changes of intestinal mucosa in patients with CD, covering the entire spectrum of mucosal injury—from normal (Marsh 0) to infiltrative stage (Marsh 1), hyperplastic (Marsh 2), and atrophic (Marsh 3 with subclasses 3a-c) [68]. Corazza–Villanacci has proposed a more simplified scoring system, consisting of three villous morphologies, but interobserver agreement is suboptimal in both [69,70,71].

Criteria to assess remission on follow-up histology were also heterogeneously defined among included studies. Some authors used standard Marsh criteria, defining remission as Marsh 0, Marsh 0–1, or Marsh 0–2 (Marsh < 3); others defined outcomes as “no villous atrophy”; others used quantitative histology (villous height to crypt depth ratio, Vh/CrD) [7,11,30,35,39,50,53,57]; and very few relied on highly accurate markers such as anti-transglutaminase-2 IgA mucosal deposits in the distal duodenum [37]. Most studies inferred remission based on Marsh 0 or 1 control biopsy, while some explicitly defined remission as Marsh 0 only, reflecting complete normalization of mucosal histology, also termed deep remission [64]. Only a few studies did not adopt the Marsh-based histology reporting, instead using the alternative Corazza–Villanacci classification system [29].

Considering the inconsistent definition of histological remission, rates of mucosal healing vary substantially across studies, closely linked to the specific criteria employed to define resolution of mucosal injury: lower values were seen when using stricter criteria such as quantitative histology (Vh/CrD)—21% in Lee et al. [30], 37% in Rubio-Tapia et al. [7]—while higher rates were reported in studies accepting only descriptive normalization of villous architecture (villous recovery, absence of VA) or partial improvement (Marsh 1–2). The difference in proportion of histologic remission rate according to criteria used is well delineated in the paper by Caruso et al. [37], where 84.6% of patients had no VA on follow-up biopsy, but only 53.8% had no TG2 mucosal deposits. Even among studies using quantitative morphometric criteria, there was a difference in the thresholds used to define remission, from Vh/CrD 4–3:1 in Cammarota et al. [35] to Vh/CrD ≥ 2.0 in Lee et al. [30].

### 3.4. Histologic Remission Rates

**Figure 2:**
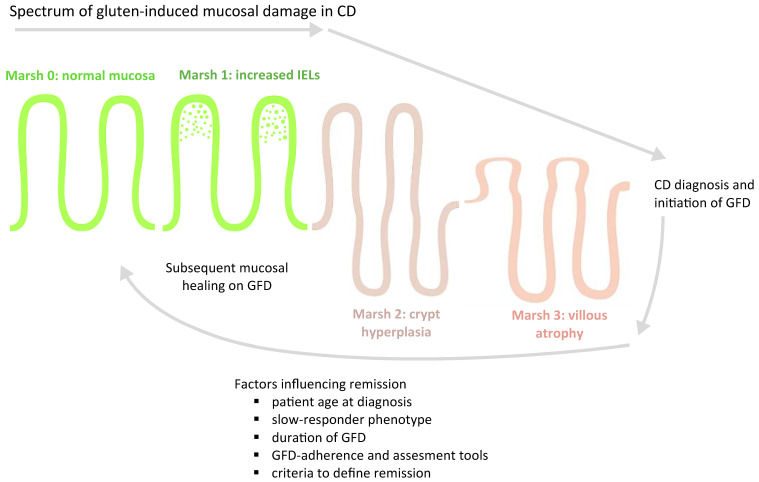
Spectrum of gluten-induced mucosal injury in CD and subsequent mucosal recovery after dietary gluten exclusion.

Histological outcomes varied widely among the studies, ranging from less than 10% to over 90%. The major factors influencing resolution rates were duration of GFD, adherence assessment tools, and, critically, the criteria to define remission, which limit the comparability across cohorts (Figure 2).

Considering histologic remission as Marsh 0–2, villous recovery, or absence of VA, a weighted-average approach was used when rates were reported for subgroups, while the rates for the longest follow-up were used in studies with several timepoint evaluations after GFD initiation. The overall mucosal recovery was seen in 58.8% of patients.

The highest remission rates were observed in prospective cohorts with rigorous adherence monitoring—weighted average 93.6% in Laurikka et al. [50] and 85% in Newnham et al. [56]. Conversely, lower remission rates were seen when using quantitative histology in the setting of clinical trials, such as the cohorts analyzed in Daveson et al. [39], which revealed VA in the majority of CD patients who appeared well controlled on GFD.

Interestingly, some authors did not find any difference in the proportion of remission rates between short-term-treated or long-term-treated patients with CD—93% (1–2 years) vs. 94% (≥3 years) in the paper by Laurikka et al. [50]—while others clearly demonstrated higher recovery rates in correlation with longer duration of GFD—30% after 6 months of GFD vs. 47.4% after 24 months of GFD [60].

Others revealed slight differences according to age—79% remission in patients ≥ 65 years vs. 82% in patients 18–65 years in the paper by Casella et al. [28].

**Figure 3:**
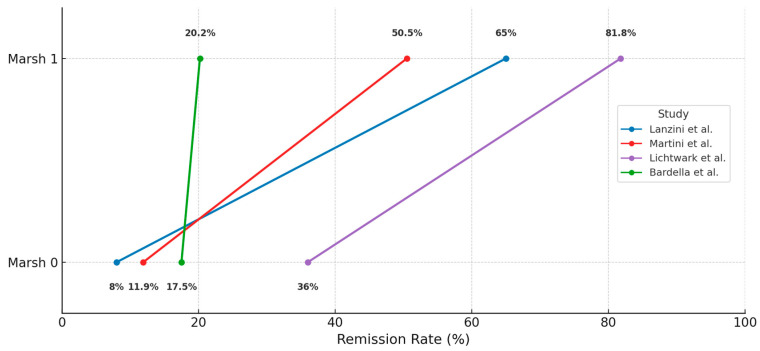
Remission rates according to Marsh 0 vs. Marsh 1 thresholds [16,46,52,54].

Several studies showed increasing rates when considering remission beyond Marsh 0, such as in the study by Lanzini et al. [46] (from 8% Marsh 0 to 65% Marsh 1), Martini et al. [54] (11.9% Marsh 0 and 50.5% Marsh 1), and Lichtwark et al. [52] (36% Marsh 0, 81.8% Marsh 0–1), while others showed no major difference, such as in the paper by Bardella et al. [16] (17.5% Marsh 0 and 20.2% Marsh 1), as shown in Figure 3.

Others have stratified rates of histologic recovery according to serology, such as Farina et al. [25], who reported paradoxical findings—VA at follow-up was observed in 10% of tTG-positive CD-treated patients vs. 36% in tTG-negative CD individuals—which reinforces the low sensitivity of serology in predicting mucosal damage. The results of Farina et al. might be due to differences in clinical phenotype, as tTG-positive CD patients were more frequently diagnosed by screening, which might be correlated with milder mucosal involvement.

### 3.5. Correlation with Clinical Symptoms and Serology

Most studies support the lack of correlation between resolution of symptoms and seroconversion with mucosal healing. The study by Duerksen et al. [42], although subject to potential bias in symptom reporting, included asymptomatic patients with CD with a mean GFD duration of 9.7 years, amongst whom only half achieved Marsh 0–1 on follow-up biopsy. In the cohort by Tuire et al. [11], of all patients having negative celiac serology, only 42% had complete histologic remission (Marsh 0), and overall 96% achieved villous recovery. Very high rates of persistent mucosal damage were seen in Daveson et al. [39], with 90% of patients with VA having negative serology.

## 4. Discussion

Histologic remission in GFD-treated individuals is a key indicator for disease outcome, being strongly correlated with complications and mortality [61,72]. While clinical features and serologic markers do not accurately predict restitution of villous architecture, the follow-up biopsy is the only instrument to document mucosal healing in CD. However, endobiopsies have certain limitations: invasiveness, sampling error, reduced patient willingness, and interobserver variability in histologic interpretation. In this setting, emerging non-invasive techniques (biomarkers, cytokines, and imaging tools) to monitor mucosal healing could complement or eventually reduce reliance on small bowel biopsies [73].

In this literature review, we collected data about adult patients with CD on GFD and revealed that most patients have persistent enteropathy despite apparent good diet adherence and negative serology. Included studies span over two decades of evolving diagnostic criteria—while in the early 1990s, diagnosis primarily relied on symptoms and small bowel biopsy, serology has since become an essential instrument for diagnosing CD, even eliminating the need for histology in certain conditions in pediatric guidelines. After the identification of tissue transglutaminase as the autoantigen in CD in 1997 [74], commercial tests for tTG were rapidly developed and validated. In more recent studies, owing to the increased availability of serologic testing, patients might have been diagnosed earlier in the disease course, with the potential for milder histologic damage at baseline and consequently more rapid histologic recovery. Furthermore, increasing access to endoscopy and refinements in endoscopic imaging have enhanced the precision of duodenal mucosal assessment and detection of subtle mucosal changes. Additionally, standardized histologic scoring systems have become more widely adopted in recent years, improving consistency in reporting compared to older studies. Moreover, histologic recovery rates over time might have been influenced by access to gluten-free products and dietary monitoring tools. A temporal trend impacting comparability of histologic outcomes relates to the follow-up protocol, with control biopsies to document mucosal healing not being carried out routinely in later cohorts but only for patients with persistent symptoms or positive serology, thus potentially underestimating histologic recovery rates.

A first challenge in the follow-up of CD is how to define histologic remission—from qualitative assessment under the terms of mucosal healing, recovery, or response to using standardized but subjective classification systems such as the Marsh score [75]. The most reliable assessment would be quantitative morphometry, as this raises the threshold for histologically normal mucosa, but this is not currently available in routine practice and is used in research settings only, hindered by the requirement of technical expertise and equipment as well as processing costs and turnaround time [20]. This heterogeneity in histological definitions poses a major challenge in comparing outcomes across studies and consequently in guiding interventions in the follow-up of CD individuals. Moreover, there are also advanced diagnostic techniques beyond conventional histology, such as transglutaminase-2-specific mucosal IgA autoantibody deposits, which are considered an accurate marker of disease activity [76]. In the rigorous setting of a clinical trial, the CeliAction Study, which comprised 1345 GFD-treated CD patients with self-reported moderate or severe CD-associated symptoms and used quantitative histology, showed only 8% of patients had normal mucosa as defined by a VH/CrD ratio ≥ 3.0 [20].

As for other chronic diseases, monitoring CD begins with symptom control, proceeding then to serological and histologic assessment. Persistence of symptoms is not uncommon in patients with CD, and the diagnostic approach of non-responsive CD includes evaluation for persistent VA [77]. While there is substantial variability in mucosal healing rates among patients with CD on GFD depending on several factors, assessment of histologic recovery is recommended after at least 1–2 years of strict dieting [78], subject to the category of slow responder patients, in whom healing may be delayed [8]. Moreover, although adherence data are variable, up to 90% [79], real-world data shows there is gluten contamination in long-term-treated patients with CD [80].

Among the factors that impact histologic remission rates in CD, there are variations in how mucosal healing is defined, GFD duration, and adherence, along with the study population and patient age at CD diagnosis. In our review, the parameter that contributed the most to heterogeneity of remission rates was the definition of mucosal recovery, which ranged from complete normalization (Marsh 0) to partial improvement of mucosal architecture with the absence of VA (Marsh 0–2), thus limiting cross-study comparisons. Studies defining remission as Marsh 0–2 on follow-up histology based on descriptive data showed higher remission rates compared to studies that required more strict Marsh 0 for control biopsy or used quantitative evaluation of villous height and crypt depth ratio.

There is a debate whether Marsh 1 is an acceptable goal for CD remission compared to complete normalization of architecture, including normal IELs (Marsh 0), as persistence of intraepithelial lymphocytosis has been associated with significantly lower (but within normal range) villous height-crypt depth ratio (2.9 vs. 3.2, *p* = 0.042) [8,11]. Our review demonstrated significant differences in remission rates when considering Marsh 1 over Marsh 0, such as in the paper by Lanzini et al. [46], which classified an additional 57% of patients as healed—from 8% Marsh 0 to 65% Marsh 1. Moreover, within a Marsh 1 histology, the distribution pattern of IELs seems to be correlated with GFD adherence [81]: patients classified as having good compliance exhibited a basal IEL pattern, while those with poor compliance showed mixed and apical patterns.

Another point clearly seen in our dataset is that neither clinical symptoms nor serology accurately predict mucosal healing, and the gold standard for persistent VA remains follow-up biopsy [42,66,82]. Notably, for the baseline biopsy, the repeat biopsy should also include bulbar specimens, which increase detection of persisting VA by 10% [83,84].

Age is also an important factor that influences histologic remission rate in CD. Due to prolonged gluten exposure during the lifespan of an adult and the diagnostic delay in this patient population, mucosal healing is slower and less complete in adults compared to children [14,16,23,85]. Given the fast and high rates of remission in children, routine re-biopsy to assess mucosal recovery is not recommended in pediatric CD [86]. On the other hand, in adults, it might be the only chance to look at mucosal architecture, especially with the emerging concept of a no-biopsy strategy in adults, where the baseline endoscopy will be dropped [87]. Currently, there is a lack of consensus with regard to the requirement of a repeat biopsy for adults, but it should be considered, especially in older adults and those with severe mucosal injury at diagnosis and seronegative CD [2]. Several studies have shown older age to be independently associated with persistent VA [45,61].

Another potentially important factor in obtaining high remission rates is education and accessibility of GFD, with studies showing lower rates of persistent VA in recent years [51].

In summary, assessment of remission is an important outcome measure in monitoring CD, as it correlates with lower complications and reduced mortality [7]. Currently, documenting mucosal healing in response to GFD by endoscopic re-biopsy is driven by the prognostic value of finding persistent VA. In the setting of a no-biopsy diagnostic protocol in adults, assessing mucosal healing might be challenging when baseline histology is not available for comparison and improvement is difficult to ascertain, especially with persistent low-grade damage in the follow-up [10]. With regard to the timing of re-biopsy, early sampling of mucosa after GFD institution can reveal a large number of patients with persistent VA, with most studies suggesting that healing should be assessed after more than 1–2 years.

The major limitation of the current work is the large heterogeneity in study design and data reporting, highlighting the need for standard outcome measures in the follow-up of adult CD patients.

## 5. Conclusions

Histologic remission in GFD-treated adult patients with CD is highly variable and strongly influenced by remission definitions and adherence assessment methods. Standardizing histologic outcome measures (scoring systems and timeline of control biopsy) and dietary compliance evaluation (using validated questionnaires and non-invasive tools) is essential for harmonizing follow-up strategies and improving clinical decision-making in adult CD. Future research should prioritize robust methodology with standardized metrics in order to accurately identify factors influencing histologic remission rate in CD and guide tailored follow-up strategies.

## Figures and Tables

**Table 1:** Studies selected from the literature search reporting histological outcomes in GFD-treated adult CD patients.

Author, Year	Population	*n*=	Age (Median/Range or Mean/SD)	Gender (% Female)	Histo at Diagnosis	Duration of GFD	Assessment of GFD Adherence	% Histologic Remission	Criteria for Histologic Remission (Mucosal Recovery)	% Serology Negative	% Asymptomatic
Bardella, 2007 [16]	Biopsy-proven CD	114	33	71.9	All Marsh ≥ 3a (Marsh 3a, 11.4%; Marsh 3b, 21.1%; Marsh 3c, 67.5%)	2 years (1–23)	Dietitian interview	17.5% Marsh 0; 20.2% Marsh 1	Normal villi, <25 IELs	100%	100%
Biagi, 2012 [34]	Biopsy-proven CD	141	34	76.6	All Marsh 3	27 months (6–298)	Biagi score	85.8%	Absence of VA	73% EMA negative	N/A
Biagi, 2014 [40]	Biopsy-proven CD + positive EMA/tTG	317	33.1 ± 12.1	74.4	All Marsh 3a-c	17 months (13–30 months)	N/A	92.1%	Marsh 0–1	76% EMA negative	70.6% asymptomatic
Cammarota, 2007 [35]	Biopsy-proven CD	62	35.1 (estimated)	83.9	All Marsh 3c	1 year	Clinical follow-up + serology	59.67%	Vh/CrD 4–3:1	79% tTG negative (49/62), 80.64% EMA negative (50/62)	N/A
Ruiz-Carnicer, 2020 [36]	CD patients on GFD for ≥24 mo	77	37	68.8	87% Marsh II-IV	≥2 years	CDAT, u-GIP	76%	Marsh 0–1	90%	77%
Caruso, 2014 [37]	Biopsy-proven CD, group C (follow-up)	13	36 (25–47)	92.3	All with VA	7 years (range 2–14)	Negative serology	84.6%	No VA, but only 53.8% negative on mucosal deposits of anti-TG2 antibody	100% EMA and TG2 antibody negative	30.8%
Casella, 2012 [28]	Biopsy-proven CD	1225	36.9 (estimated)	73.7	81.8% Marsh 3 in group A (≥65 y), 88.1% in group B (18–65 y)	30 months	Physician-based interview, Likert scale	79% group A, 82% group B	Normal villous architecture reconstituted	82% group A, 83% group B—tTG negative	45% group A; 62% group B
Ciacci, 2002 [38]	Biopsy-proven CD on GFD for ≥2 years	390	34.8	76.7	95.3% Marsh ≥ 3a	6.9 ± 7.5 years (2–22)	Structured dietary interview	43.6%	Marsh 0	75.1% EMA negative	N/A
Cuoco, 1998 [41]	Biopsy-proven CD	23	32.5 (18–42)	69.6	All Marsh 3	12 months	Direct patient questioning	84.6%	Absence of VA	92.3% EMA negative	84.61% symptom-free
Daveson, 2020 [39]	Biopsy-proven CD on GFD ≥ 1 year	93	40	71	Marsh 3 at diagnosis	6 years	CDAT	39% Marsh 0–2, 6% Marsh 0–1, 33% Marsh 2	Vh/Crd ≥ 2.8 for Marsh 0	84% TG2 IgAb negative	N/A
Dickey, 2000 [43]	Biopsy-proven CD (Marsh criteria) + serology (IgA EMA at 3, 6, and 12 m after the diagnosis)	53	51 (16–81)	73.6	All Marsh 3	1 year	Dietitian dietary review	24.5% Marsh 0	Marsh 0	87% EMA negative at 12 months	N/A
Duerksen, 2010 [42]	Biopsy-confirmed or serology-confirmed CD	22	50.5	86.4	91% biopsy-proven CD	9.7 years (1.3–50)	3-day food diary	57.14%	Marsh 0–1	N/A	100%
Elli, 2015 [44]	Biopsy-proven CD, repeat biopsy after GFD ≥ 1 year	69	39 ± 15	76	All Marsh 3 (72% Marsh 3c)	4 ± 3 (range 1–13)	Clinical follow-up	29% Marsh 0, 46.3% Marsh 0–2	Absence of VA	N/A	N/A
Fang, 2017 [29]	Adult CD patients on GFD, all with negative tTG IgA serology	402	50.3 ± 16.5	72.1	Marsh grade not specified	59.8 ± 59.4 months	Provider notes	42.3%	Normal Corazza–Villanacci histology, no VA, <25 IELs	all tTG IgA < 4 U/mL	40.5% (estimated)—51.8% of those with normal biopsy (*n* = 170) and 32.3% of those with abnormal biopsy (*n* = 232)
Farina, 2021 [25]	Diagnosis of CD according to international guidelines (ESsCD 2019, AJG 2013, ESPGHAN 2012)	277 (65 CD-treated with positive tTGA on follow-up, 212 CD-treated with negative tTGA)	38.5 (estimated)—37 (14–86) in tTGA+, 39 (16–78) in tTGA-	83.4 (estimated)—88 in tTGA+, 82 in tTGA-	N/A	4 years (1–26)	Clinical interview, CDAT questionnaire, urinary GIP	70.1% (estimated)—90% remission in tTGA-positive group vs. 64% in tTGA-negative group	Absence of Marsh 3 on follow-up endoscopy	N/A	N/A
Fernández-Bañares, 2021 [45]	Biopsy-proven CD	76	36.5 ± 1.6	73	All Marsh 3	2 years	Standardized dietitian assessment, using dietary and food label quiz, Likert scale, and f-GIP testing	47%	Marsh < 3	75%	72.5%
Galli, 2014 [32]	Biopsy-proven CD + serology (anti-tTG/EMA IgA)	65	38 (18–70)	72.3	All Marsh 3a–c	1 year	Biagi score	66%	Marsh 0	70.2% (estimated)—70.3% in the ADA group, *n* = 53, and 70% in the IADA group, *n* = 12	72.3% (estimated) well-being—45/53 (85%) ADA group, 2/12 (16.7%) IADA group
Hære, 2016 [48]	Biopsy-proven CD with VA	127	55 ± 14	62	VA (Marsh ≥ III)	8.1 years (2.3–22.3)	CDAT	81% mucosal healing (Marsh 0), 94% mucosal recovery (Marsh 0–2)	Marsh 0 for healing, Marsh 0–2 for recovery	93.7%	Not directly quantified, symptom score evaluated by GSRS-IBS
Hopper, 2008 [47]	Biopsy-confirmed CD on GFD > 1 year	48	52.7	68.8	All Marsh 3	>1 year	N/A	66.7–43.8% Marsh 0, 12.5% Marsh 1, 10.4% Marsh 2	Absence of VA	N/A	N/A
Hutchinson, 2010 [49]	Biopsy-proven CD	284	44.6 (32.3–57.7)	71	90.5% Marsh 2–3	1.6 years	Self-reported	35.2% complete histopathological recovery, 79.9% histological improvement	Histopathological disease score based on modified Marsh grade	N/A	N/A
Kaukinen, 2002 [31]	Biopsy-proven CD	87	49	72	All Marsh 3	1 year (1–18)	Dietitian interview + 3 day food diary	69% (estimated)	Marsh 0–2 considered recovery	N/A	N/A
Khurana, 2023 [27]	positive serology and/or duodenal biopsy confirmation	126	50.5 (estimated)	66.7	98.41% VA	3 months	Indirect, based on symptom improvement and dietitian referral (52,9%)	47,6% (10/21 with follow-up biopsy had normal histology)	Normal histology	54.43%	79,2% were without abdominal pain; other symptoms improved variably
Lanzini, 2009 [46]	Biopsy-proven CD	465	31 (18–81)	76.7	92% Marsh 3; 6% Marsh 2; 2% Marsh 1	16 months	Likert scale	8% Marsh 0, 65% remission with persistent IEL	Marsh 0	87% with negative CD-related serology	69.2% (estimated)
Laurikka, 2016 [50]	Biopsy-proven CD	856 (128 untreated, 93 short-term-treated CD, 635 long-term-treated CD)	54 (15–85)	75	VH/CrD < 2.0	1–2 y GFD in *n* = 93, ≥3 y GFD in *n* = 635	Dietary interview + an objective estimation (EmA positive if >2 years on GFD considered non-adherence)	93% short-term-treated, 94% long-term-treated mucosal recovery	Vh/CrD ≥ 2.0	8% EmA positive in short-term-treated CD, 3% EmA positive in long-term-treated CD	N/A
Lebwohl, 2014 [51]	Biopsy-proven CD-VA (Marsh 3)	7648	27	63	Marsh 3	1.3 years	Not measured directly	57% mucosal recovery	Marsh < 3	59% seronegative at the time of their follow-up biopsy (serology data available for a subset of patients)	N/A
Lee, 2003 [30]	Biopsy-proven CD	39	52	63	All Marsh 3	8.5 years (1–45)	Physician assessment	21% Marsh 0	Vh/CrD 4:1	77% negative serology	N/A
Leong, 2008 [58]	Known or suspected CD	17	41	71	76% histopathology changes	1 year	Accredited dietitian assessment	36.36%	Marsh 0	N/A	N/A
Lichtwark, 2014 [52]	Biopsy-proven CD	11	33	73	81.8% Marsh 3	12 months	Food diaries	36% Marsh 0, 81.8% Marsh 0–1	Marsh 0 (mucosal remission) or 1 (response)	64%	N/A
Mahadev, 2017 [53]	CD on GFD > 1 year	1345	46	81	N/A	4	Not assessed	62%	VH/CD > 2	N/A	0% (inclusion criteria—symptomatic pts)
Martini, 2002 [54]	Biopsy-proven CD	101	37 (21–72)	78.2	94% Marsh 3	1 year ± 1 month	N/A	11.9% Marsh 0, 50.5% Marsh 1	Marsh 0	N/A	N/A
McMillan, 2001 [55]	Biopsy-proven CD	36	26–76	72.2	All Marsh 3	12 months ± 2	Dietitian review + food diaries	61.1% improved histology	Marsh 0–1	N/A	N/A
Newnham, 2016 [56]	Adult CD: Marsh ≥ 2 with positive serology and HLA-DQ2/8+	99	40	76	Marsh ≥ 2	Longitudinal assessment at 1 year/5 years	Dietitian interview	37% Marsh 0 and 54% Marsh 0–1 at 1 year, 50% and 85% at 5 years, respectively	Marsh 0	70% TTG IgA negative at 5 years	N/A
O’Keeffe, 2001 [26]	Biopsy-proven CD (typical histological lesion)	12	37 (estimated)	75	All Marsh 3	3 years (range 2 months−7 years)	N/A	50%	Normal histology	91.7%	N/A
Packova, 2020 [59]	CD on a GFD ≥ 1 year + follow-up biopsy and serology	82	33.8 ± 17.4	81.7	All pts Marsh 2–3 (2 Marsh 2, 17 Marsh 3a, 30 Marsh 3b, 33 Marsh 3c)	85.4% ≥ 2 years	Experienced dietitian	76.8%	Marsh 0–1	77.8% aTTG negative; 62.8% aDGP negative	76.8% had no diarrhea, 79.3% no abdominal pain
Pekki, 2015 [57]	Biopsy-proven CD + GFD 1 year	263	45	68	All patients Vh/CrD < 2.0	1 year	Dietitian interview	68%	Vh/CrD > 2	89% in histologically recovered; 84% in atrophy group	N/A
Rubio-Tapia, 2010 [7]	Biopsy-proven CD	241	47 (18–84)	73	99.17% Marsh 3	≥5 months on GFD	Dietitian interview	37% mucosal recovery at first follow-up biopsy, 45% histological improvement	Vh/CrD ≥ 3	66% tTG negative, 81% EMA negative at follow-up biopsy	82% pts clinical response
Sadeghi, 2020 [60]	Biopsy-proven CD (Marsh III)	58	39.5 ± 13.7	56.8	All Marsh III	Group A (6 months): 20, group B (24 months): 38	Validated structured 4-question dietary adherence questionnaire	30% group A (6 mon GFD), 47.4% group B (24 months GFD)	Marsh 0	75%– group A, 78.9– group B	50– group A, 57.9–group B
Sategna-Guidetti, 2000 [62]	Biopsy-proven CD	86	29 (19–67)	74.4	Marsh classification not mentioned	1 year	Not formally assessed	56.9% mucosal recovery	Not specified	N/A	N/A
Schiepatii, 2023 [61]	Biopsy-proven CD	694	44 ± 16	70.7	All Marsh ≥ 3a	32 months (IQR 15–61)	Dietetic interview or validated questionnaires (CDAT/Pavia score)	77.40%	Marsh < 3a on follow-up biopsy	N/A	65.9% symptom improvement
Selby, 1999 [63]	Biopsy-proven CD	89	47.2 ± 13.6	82	VA	8.3 ± 6.7 years (range, 0.6–29.2 years)	Dietary interview, food diary, questionnaire	57%	Absence of VA	N/A	97.3% EMA negative
Sharkey, 2013 [67]	Biopsy-proven CD	595 (adults + children)	46	70.8 (whole cohort)	Marsh 3	11 months	Dietitian review	30%	Marsh 0–2 = recovery	N/A	N/A
Silva, 2020 [64]	CD diagnosis according to 2016 WGO guidelines	69	22.5	79.7	75.5% Marsh ≥ 3	7.98 ± 5.6 years	N/A	37.7% Marsh 0, 40.6% Marsh 1	Marsh 0	N/A	N/A
Tuire, 2012 [11]	Biopsy-proven CD, GFD ≥ 2 years	177	55.7 (estimated)	72.9 (estimated)	Marsh 3	9.5 (estimated)	Dietitian interview	96% normal villous architecture, 42% Marsh 0	Vh/CrD ratio	100%	N/A
Tursi, 2006 [65]	Biopsy-proven CD	42	32.7	69	80.95% (34/42) Marsh ≥ 3a	2 years	Structured interview	59.5% Marsh 0	Marsh 0	N/A	N/A
Vahedi, 2003 [33]	Biopsy-proven CD + serology (EMA/tTG)	95	41 (17–74)	73.7	Marsh 3	75 months (12–398)	Dietitian assessment	65%	Normal villous architecture	97.5% EMA negative in strict adherents	N/A
Vécsei, 2009 [66]	Biopsy-proven CD	47	45 (16–74)	66	Marsh ≥ 3	Group A: ≤2 years (median 15 months); Group B: >2 years (median 40 months)	Physician assessment	57.5%	Villous recovery (Marsh 0–2)	N/A	N/A

Abbreviations: CD—celiac disease, EMA—endomysial antibodies, tTG—tissue transglutaminase antibodies, TG2—transglutaminase 2, DGP—deamidated gliadin peptides, GFD—gluten-free diet, IEL—intraepithelial lymphocytes, VA—villous atrophy, CDAT—Celiac Dietary Adherence Test, GIP—gluten immunogenic peptides, u-GIP—urinary GIP, f-GIP—fecal GIP, Vh/CrD—villous height to crypt depth ratio, HLA—human leukocyte antigen, ESsCD—European Society for the Study of Coeliac Disease, AJG—American Journal of Gastroenterology, ESPGHAN—European Society for Pediatric Gastroenterology, Hepatology, and Nutrition, WGO—World Gastroenterology Organization, N/A—not available.

## Data Availability

Data from the original articles included in the review is available from the corresponding publisher.

## Supplementary Materials

The following supporting information can be downloaded at https://www.mdpi.com/article/10.3390/jcm14145144/s1. The search strings used for the literature review are available as Supplementary File S1.

## Abbreviations

The following abbreviations are used in this manuscript:

CD	Celiac disease
GFD	Gluten-free diet
VA	Villous atrophy
VH	Villous height
CrD	Crypt depth
IEL	Intraepithelial lymphocytes
EMA	Endomysial antibodies
tTG	Tissue transglutaminase antibodies
TG2	Transglutaminase 2
DGP	Deamidated gliadin peptides
CDAT	Celiac Dietary Adherence Test
GIP	Gluten immunogenic peptides
HLA	Human leukocyte antigen
