# Supplementary material for: Outcomes in Adults with Celiac Disease Following a Gluten-Free Diet
Source: J Clin Med. 2025 Jul 20;14(14):5144. doi: 10.3390/jcm14145144 (PMC12294898; doi:10.3390/jcm14145144)
Supplement: Supplementary file 1 [file jcm-14-05144-s001.zip › jcm-3725199-supplementary.pdf]

Supplementary File S1 - Search strings used for the literature review

Pubmed search string
("celiac disease"[MeSH]) AND ("long-term" OR "treated") AND ("gluten free diet" OR "GFD") AND ("remission" OR "follow-up")

Scopus
TITLE-ABS-KEY("celiac disease") AND (TITLE-ABS-KEY("long-term") OR TITLE-ABS-KEY("treated")) AND (TITLE-ABS-KEY("gluten free diet") OR TITLE-ABS-KEY("GFD")) AND (TITLE-ABS-KEY("remission") OR TITLE-ABS-KEY("follow-up"))
